# Do Telomeres Adapt to Physiological Stress? Exploring the Effect of Exercise on Telomere Length and Telomere-Related Proteins

**DOI:** 10.1155/2013/601368

**Published:** 2013-12-24

**Authors:** Andrew T. Ludlow, Lindsay W. Ludlow, Stephen M. Roth

**Affiliations:** ^1^Department of Cell Biology, UT Southwestern Medical Center, Dallas, TX 75309, USA; ^2^Department of Applied Physiology and Wellness, Southern Methodist University, Dallas, TX 75206, USA; ^3^Department of Kinesiology, School of Public Health, University of Maryland, College Park, MD 20742-2611, USA

## Abstract

Aging is associated with a tissue degeneration phenotype marked by a loss of tissue regenerative capacity. Regenerative capacity is dictated by environmental and genetic factors that govern the balance between damage and repair. The age-associated changes in the ability of tissues to replace lost or damaged cells is partly the cause of many age-related diseases such as Alzheimer's disease, cardiovascular disease, type II diabetes, and sarcopenia. A well-established marker of the aging process is the length of the protective cap at the ends of chromosomes, called telomeres. Telomeres shorten with each cell division and with increasing chronological age and short telomeres have been associated with a range of age-related diseases. Several studies have shown that chronic exposure to exercise (i.e., exercise training) is associated with telomere length maintenance; however, recent evidence points out several controversial issues concerning tissue-specific telomere length responses. The goals of the review are to familiarize the reader with the current telomere dogma, review the literature exploring the interactions of exercise with telomere phenotypes, discuss the mechanistic research relating telomere dynamics to exercise stimuli, and finally propose future directions for work related to telomeres and physiological stress.

## 1. Introduction

Broadly, aging is defined as the accumulation of cellular damage that results in a loss of cellular and organismal fitness. Aging is marked by a substantial decrease in the regenerative potential of several cell types, including immune cells and skeletal muscle cells [1, 2]. Both genetic and environmental factors dictate the rate of tissue regeneration and the balance between accumulation and removal of cellular damage. Accumulation of unrepaired cellular damage and a lack of tissue regeneration via cell replication result not only in aging-related phenotypes (grey hair, wrinkled skin, etc.), but also in several age-related diseases such as Alzheimer's disease, cardiovascular disease, type II diabetes, and sarcopenia [3, 4]. Aging impacts the genome in several ways. For example, aging modifies the structure-function relationship of the genome through accumulation of mutations, changing epigenetic profiles (both changes in DNA methylation patterns and histone modification) and altered telomere dynamics [5, 6]. How physical activity and exercise training can modify the age-associated genomic changes is beginning to be explored and thus far has produced exciting results.

Interestingly, all of the aforementioned age-related diseases are modified by physical activity. Further, recent evidence indicates that telomere length is also associated with several age-related diseases, and that telomere length and the suite of proteins that maintain telomere length are altered by changes in physical activity level [7]. Thus, the purpose of this review is to describe the basic biological implications of telomeres and telomere shortening, to explain the function of the suite of telomere-associated proteins, and to review the recent literature involving telomere length and telomere-associated proteins as they are affected by exercise training or physical activity. We further discuss the potential mechanisms of how exercise may cause beneficial adaptations in the telomere length maintenance system.

## 2. Human Telomere Biology

**Figure 1:**
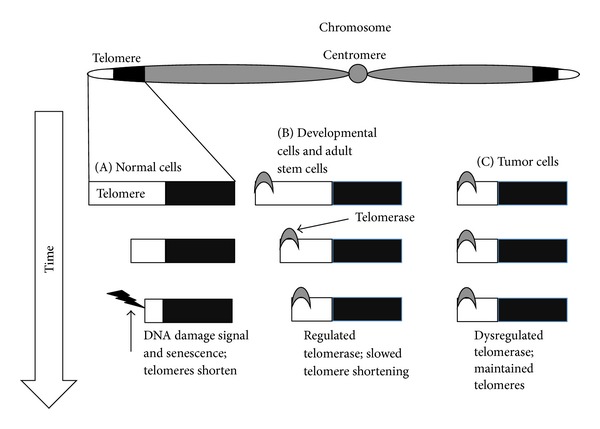
Common telomere/telomerase dogma across cell types in humans. Telomeres are located on the ends of linear chromosomes. Over time (i.e., with increased numbers of cell divisions), telomeres shorten due to a number of end-processing events; thus, short telomeres are associated with chronological age and a number of age-related diseases. (A) Telomeres function to mask the ends of chromosomes from being recognized by a cell's DNA damage response system. When telomeres reach a certain length, they are no longer masked and the cell recognizes the ends of the chromosome as damaged DNA. When the DNA damage signal is initiated, the cell arrests and enters telomere-induced senescence. This occurs in adult human cells lacking the enzyme telomerase, which maintains and elongates telomeres by using reverse transcriptase activity to add telomere repeats to the ends of chromosomes. (B) During development and in certain adult stem cells, telomerase is expressed and slows telomere shortening in these cells, thus maintaining the pool of cells available in a presenescent state. (C) In 85% of tumor cells, telomerase is dysregulated and allows cancer cells to be immortal and divide indefinitely since they do not undergo telomere-driven senescence.

Recently, short telomeres have become a widely accepted molecular/cellular hallmark of aging [8]. Telomeres are repetitive DNA sequences (5′-TTAGGG_*n*_-3′) at the ends of linear chromosomes [9]. With each cell division, telomeres shorten by about 60 base pairs due to the inability of DNA polymerase to fully replicate the chromosome end, a phenomena referred to as the end-replication problem (Figure 1; [10, 11]). In this sense, telomeres act as a mitotic clock that “records” the number of divisions a cell has undergone. When telomeres shorten to a critical length, the chromosome ends are recognized as DNA-double-strand breaks by the DNA damage response system [12–15]. Therefore, the second function of the telomere is to prevent a DNA damage response at the chromosome end through maintenance of a sufficient length, subsequently solving the “end-protection problem” [13, 14]. Thus, critically short telomeres in aged cells may be recognized as DNA double-strand breaks, ultimately causing the cell to enter senescence (Figure 1; [14, 16–21]). An increase in senescent cells, which may be observed in response to critically shortened telomeres, is a major part of tissue dysfunction with aging and is associated with age-related phenotypes (Figure 1; [22–24]). While senescence may occur via nontelomeric mechanisms, accumulation of replicative senescent cells (i.e., telomere-driven senescence) in tissues significantly contributes to the aging process.

Repeated cell division is one mechanism for reduced telomere length; however, other mechanisms also contribute to the total rate of telomere shortening in cells [25]. For example, chronic exposure to DNA damaging agents (e.g., UV, oxidative stress, and inflammation) may result in a sudden loss of large amounts of telomere DNA [26]. Therefore, telomeres are shortened by a combination of chronic, gradual shortening due to repeated cell division and acute, stochastic shortening of a few or single telomeres in a cell caused by DNA damaging agents.

Certain cell types, even with continuous cell division, are able to maintain their telomeres despite the end-replication problem. In highly mitotic cells (i.e., germline cells, stem cells, specialized subsets of immune cells, and cancer cells), the gradual telomere shortening due to the end-replication problem is overcome by the enzyme telomerase (Figure 1; [10]). In cells with telomerase, telomere length is maintained with each cell division and thus these cells are essentially immortal and able to undergo many population doublings without the accumulation of short telomeres (Figure 1). Therefore, the present review will focus not only on telomere length, but also on the suite of telomere-binding proteins in immune cells, skeletal muscle, and other tissues impacted by physical activity or exercise.

### 2.1. Proteins Involved in the Regulation of Human Telomere Length: Telomerase and Shelterin

**Figure 2:**
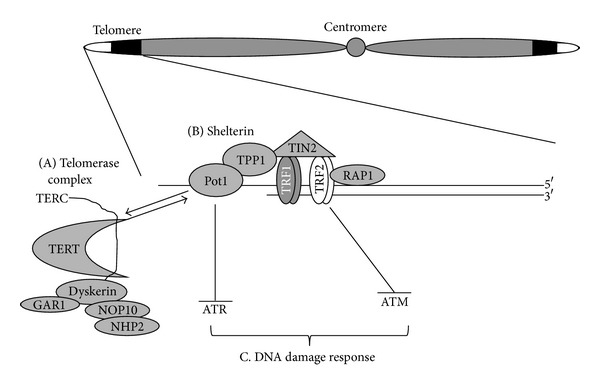
Telomere-related proteins. Telomere DNA sequences are bound by and interact with several proteins. These proteins and the enzyme telomerase function to regulate telomere length and prevent inappropriate recognition of telomere DNA by the DNA damage response machinery. (A) Telomerase is a ribonucleoprotein consisting of two core components: a catalytically active reverse transcriptase component, TERT and a noncoding RNA template, TERC. Together with several other cofactors such as dyskerin, GAR1, NOP10, and NHP2, telomerase functions to add telomere repeats to the ends of telomeres. (B) A complex of six proteins termed “shelterin” binds to telomere DNA in a tightly regulated stoichiometry and functions to regulate telomere length by preventing inappropriate telomere elongation by telomerase. Telomere repeat binding factors (TRFs) 1 and 2 bind to telomere double-stranded DNA and function to regulate telomere length and T-loop formation. (C) Shelterin also functions to prevent the DNA damage machinery from recognizing telomeres. Both POT1 and TRF2 prevent the telomere from being recognized by DNA damage kinases.

Telomerase is a ribonucleoprotein that consists of two central components: a protein reverse transcriptase component (TERT) and an RNA template (TERC) [6, 27, 28]. From a functional standpoint, telomerase is thought to be preferentially recruited to short telomeres [29–33]. Current dogma is that telomerase activity is confined to developing cells, adult stem cells, germline cells, and subsets of immune cells and that most somatic tissues have low or undetectable telomerase; thus, telomerase in healthy adult tissues is tightly regulated (Figure 2). However, in cancer tissues telomerase activity is dysregulated, and about 85% of cancers/tumor cells have detectable telomerase activity, thus allowing cancer cells to maintain their telomeres despite regular cell division (Figure 2; [34]). In addition to enzymatic activity, telomerase processivity (i.e., a special property of telomerase that describes the ability of telomerase to successively add TTAGGG repeats to telomere ends) depends on several factors, the most important being the proteins of the telomere binding protein complex called shelterin.

Shelterin acts as both a positive and negative regulator of telomere length [13, 35] and as a negative regulator of telomerase enzyme activity [36]. Shelterin protein components bind double- and single- stranded telomere DNA and regulate telomere length by controlling the access of telomerase to telomeres [36]. In addition, shelterin also helps to solve the end-protection problem by masking the ends of chromosomes from being recognized as DNA double-strand breaks and by preventing DNA damage sensing kinases from accessing telomere ends (Figure 2; [13]). Lastly, shelterin folds the telomere DNA into a three-dimensional structure called a T-loop [37], which is thought to both control access of telomerase to telomeres and to package the telomeres into condensed heterochromatic DNA.

Shelterin consists of six proteins: telomere-repeat binding factors (TRF) 1 and 2, protection of telomeres (POT1), RAP1, TPPI, and TIN2 (Figure 2; [12, 13, 38]). TRF1 and TRF2 bind to the double-stranded portion of telomeres and dictate the state of the telomere end in either an open (telomerase accessible) or closed (telomerase inaccessible) state [19]. TRF1 and TRF2, along with their interacting proteins, RAP1 and TIN2, are important in the regulation of telomere length, as shown from gain and loss of function studies [14, 19, 39]. POT1 binds to the single-stranded portion of telomeres and is important in preventing telomere end recognition as damaged DNA and also in controlling access of telomerase to telomeres [36]. In addition, POT1 and its interacting partner TPPI have been shown to control the processivity and recruitment of telomerase to telomere ends [40]. Importantly, altering the function and/or abundance of any of the telomere-binding proteins affects telomere length regulation and function (i.e., prevention of a DNA damage response at telomeres). Thus, if physiological stress is able to alter either the function or the abundance of these important proteins, this may provide a mechanism by which exercise regulates telomere length.

Recent evidence has indicated that the environment can impact telomeres and the suite of proteins (i.e., telomerase, shelterin) that are related to telomere maintenance. Physiological stressors such as lifestyle choices and psychological stress have been shown to influence telomere length and telomerase enzyme activity [7, 41–45]. This review will focus on physiological stress in the form of physical exercise and the signals that may impact telomere biology in immune and skeletal muscle tissues.

## 3. Environmental Effects on Telomere Biology: Exercise and Physical Activity

Regular physical activity and exercise training (both resistance exercise and endurance exercise) are known to reduce the risk of developing many age-related chronic diseases such as cardiovascular disease, certain cancers, type II diabetes, and sarcopenia [7]. Exercise-related improvements in function at the whole body level are well-known, but the cellular, molecular, and genetic underpinnings are only beginning to be elucidated [46, 47]. While several groups have focused on how genetic variation influences both the response to exercise and the propensity to engage in exercise, few groups have investigated the impact of exercise on the genome structure itself (e.g., telomeres). Previous literature has described the association of various environmental stressors and lifestyle factors with telomere length, including psychological stress [44, 45, 48], comprehensive lifestyle changes (exercise and psychological stress counseling) [49, 50], diet [51, 52], body mass [53], socioeconomic status [54], and smoking status [55]. Since regular physical activity and exercise are well known environmental stressors with beneficial health outcomes (i.e., increased antioxidant gene expression, reduced inflammation, etc.), several groups have investigated the role of physical activity and exercise in human telomere biology. The notion that exercise reduces the impact of aging is well established; however, how exercise directly impacts telomere length remains to be fully elucidated. Several groups have hypothesized that exercise may be slowing cellular aging by reducing the rate of age-associated telomere shortening. However, this hypothesis is complicated by several lines of evidence that have demonstrated telomere shortening in response to extreme amounts of exercise. Here, we review how telomere length responds to exercise in both humans and rodents, point out current controversial issues in the field, and discuss data that demonstrate that telomere length may respond to exercise in a tissue-specific fashion.

We performed a systematic review of the relevant literature using the NCBI PubMed database. The following search terms were used: telomere length and exercise (45 results), telomere length and physical activity (74 results), telomerase and exercise (21 results), and telomerase and physical activity (82 results; all results current as of November 15, 2013). Studies were included in the review if they met the following criteria: (1) measured telomere length (by any method); (2) determined physical activity status either by physiological measure (i.e., maximal oxygen consumption) or survey (i.e., determination of exercise history based on questionnaire). The final number of studies included in the review concerning telomere length and physical activity/exercise was 23 (summarized in Table 1).

### 3.1. Human Telomere Length Response to Chronic Exercise Training

Telomere length changes slowly (e.g., years) and for this reason very few well-controlled exercise training intervention studies have been performed in humans. The majority of the research on human telomere length and exercise has been done retrospectively on banked DNA samples from immune cells [7, 56]. Since the majority of the studies to date have not been performed on specific subsets of immune cells, or even isolated peripheral blood mononuclear cells (PBMCs), we will refer to immune cells (leukocytes) broadly in the following section (refer to Tables 1(a), 1(b), and 1(c) for details on cell types); this is a key limitation of the existing literature.

Multiple cross-sectional studies have described the associations between physical activity and/or exercise training and telomere length in immune cells, with three different relationships reported: a positive association, no association, and an inverted U relationship. In the inverted U relationship, sedentary individuals and extremely active individuals have shorter telomeres than moderately active individuals. The cross-sectional nature of these studies, their small sample size, variation in collection of exercise and physical activity data, methods of telomere length determination, cell types used for DNA extraction, and the various ages of the individuals in the study cohorts likely explain the discrepant results.

### 3.2. Studies Showing a Positive Relationship between Physical Activity and Telomere Length

Several studies have reported a positive association between physical activity and telomere length, in that active individuals have longer telomeres in immune cells compared to sedentary individuals [45, 57–60]. Cherkas et al. [61] reported a positive association between increasing physical activity and longer telomeres, with differences in telomere length equating to about 10 years of biological age difference between active and inactive subjects [61]. Other groups have confirmed or extended these results by showing that telomere length was longer in individuals with higher maximal oxygen consumption values compared to those with lower maximal oxygen consumption values [58, 62, 63]. Krauss et al. [64] found that individuals with low exercise capacity (as measured in METS) had a greater likelihood of having short telomeres compared to individuals with a greater exercise capacity and that this difference in telomere length was equivalent to about 4 years of biological age. Further, in a study of ultramarathon runners, longer telomeres were observed in the runners compared to sedentary age-matched individuals, with the difference approximately equal to 16 years of reduced biological age [65].

### 3.3. Studies Showing No Difference in Telomere Length between Active and Sedentary Individuals

Studying a group of marathon runners compared to sedentary age- and sex-matched individuals, Mathur et al. [66] found no association between maximal oxygen consumption or physical activity level and telomere length despite an extreme difference in fitness. Several other studies have similarly reported no association between telomere length and physical activity level, but the age of the subjects, extent of physical activity, measurement of telomere length, and other uncontrolled factors (e.g., diet and psychological stress) likely contributed to the lack of association in these studies [50, 67–69].

### 3.4. Studies Showing an Inverted U Relationship between Activity and Telomere Length

A few studies have described an inverted U relationship between physical activity and telomere length where moderately active individuals exhibit longer telomeres compared to both sedentary and extremely active individuals. Ludlow et al. [43] showed that 50–70-year-old individuals in both the lowest (<990 kcal/wk) and highest quartiles (>3541 kcal/wk) of exercise-specific energy expenditure had shorter telomeres than individuals in the second quartile (991–2340 kcal/wk), even when controlling for age, gender, and body weight. Savela et al. [75] found that individuals who reported moderate levels of physical activity in midlife had a longer mean telomere length and also a smaller proportion of short telomeres compared to both low active and highly active individuals. Telomere lengths in a single cell are heterogeneous across chromosome ends and it is believed that the shortest telomere in a cell drives the induction of senescence; therefore, it is important to monitor the shortest telomere length in a population of cells [77, 78]. This is important because it takes into account the proportion of the shortest telomeres, which are likely the most important in dictating cellular fates, whereas previous reports only focused on mean telomere length.

Several factors must be considered when interpreting the mixed associations between telomere length and exercise/physical activity levels. Specifically, sampling bias, cell type, age of individuals when measures were made, and the timing of sample collection may all influence study outcomes. For instance, because circulating stem cells are released following exercise [79–81], if an active subject had recently completed an exercise bout, the peripheral blood would be biased by stem cells with longer telomeres. Thus, a 48 hr “washout” period or cell sorting techniques should be considered to prevent this type of sampling bias. Further, the inverted U phenomenon may be age dependent and only evident in older, highly active individuals, while in younger endurance athletes telomere length may be preserved even when extreme amounts of exercise are performed.

Another important factor that should be considered is the method of telomere length determination [82]. Telomere lengths in the above studies were mainly determined by two methods: terminal restriction fragment southern blot analysis or TRF and the telomere repeat copy number to single copy gene copy number ratio performed as a quantitative real-time PCR assay (qPCR). The TRF method is a southern blotting method and results in determination of mean telomere length from a smear of telomere signal [83]. Several biases may occur in this method including loss of short telomere signal and inclusion of subtelomeric DNA [82]. The qPCR method is a determination of relative telomere length that is dependent upon the ratio of telomere DNA content to chromosomal (single copy gene) DNA content within a given sample [84]. The qPCR method is highly correlated with the southern blot method, but certain differences and biases may arise. For example, the qPCR method is subject to wide variability between labs and sample preparations [85]. Nonetheless, both methods are valid for determining telomere length, but make comparisons between labs and studies difficult. Thus, one must interpret the findings of studies with these methodological issues in mind.

While the current research findings are divergent, the overall consensus from these studies is that moderate levels of physical activity are associated with longer telomere length in immune cells. To expand upon these findings, future studies should focus on how moderate exercise can maintain the shortest telomeres and not just mean telomere length in leukocytes. Cross-sectional studies have provided several key insights, but the limitations of these types of studies have produced mixed results that may be biased by sample collection, cell type utilized, and telomere length measurement methodology.

Randomized control clinical exercise trials would eliminate some of the bias associated with cross-sectional studies and help to clearly define the effects of exercise (both acute and long-term training) on telomere length and telomere biology. However, to date few longitudinal studies investigating the relationship between telomere length and exercise have been performed in humans. Shin et al. [86] exercise trained women (treadmill walking and running) for 6-months and observed no change in immune cell telomere length from baseline. This study, though interesting, has several shortcomings including small sample size and short training duration, which make the data difficult to interpret. Other longitudinal studies have produced mixed results regarding the relationship between exercise and telomere length, with most studies being of short duration (i.e., less than 1 year) and reporting no change in telomere length [45, 50, 67].

Recently, Ornish et al. [49, 50] completed a trial with three-month and five-year follow-up time points investigating the influence of environmental factors on telomere biology. The trial included comprehensive lifestyle changes (i.e., psychological stress counseling, dietary modification, increased physical activity, and social support groups) in a group of older men. No change in telomere length was observed at the three-month time point [50]; however, at the five-year followup, longer telomere length in PBMCs was observed in the lifestyle intervention group compared to controls [49]. These data provide support for the idea that exercise, in combination with other lifestyle factors (i.e., stress reduction and dietary modifications) over a five-year period is able to slow cellular aging, as indicated by reduced telomere shortening. The studies described above provide the necessary preliminary data to pursue large-scale intervention studies of the effect of exercise alone on telomere biology.

Very few studies have investigated the effects of acute exercise (i.e., single or a few bouts of exercise) on telomere length. Endurance exercise, specifically marathon running, is a potent immune cell proliferative stress [87] and causes skeletal muscle remodeling (i.e., proliferation of muscle precursor cells [88]); thus, one could hypothesize that telomere length changes could occur following marathon running. To obtain insights into this hypothesis, Laye et al. [74] measured telomere length in individuals before and after they ran 7 marathons in 7 days. No change in either leukocyte telomere length or skeletal muscle telomere length was observed. These data indicate that in trained individuals, a massive amount of acute exercise is not sufficient to cause rapid proliferation-related or stochastic DNA damage-associated telomere shortening. Moreover, these data indicate that there is a gap in the literature concerning what would happen to immune cells or skeletal muscle cells from an untrained person exposed to similar physiological stress. Future studies will be needed to clarify the role of acute exercise on telomere dynamics.

The consensus from the above studies in immune cells is that telomere length decreases with age in sedentary individuals, longer telomeres are observed in individuals who are moderately active (threshold to be empirically determined), and extreme long-duration endurance training for an extended portion of one's lifetime may result in telomere shortening. These divergent responses are likely directly related to the antioxidant capacity of the immune cells and the proliferative demand placed on the progenitor cells by the exercise stimulus.

## 4. The Curious Case of Skeletal Muscle Telomere Biology in Humans

Skeletal muscle has unique telomere biology when compared to other tissues. Skeletal muscle consists of a syncytium of multinucleated muscle fibers that are postmitotic; thus, telomere length should remain stable in this population of nuclei, with the rare exception of DNA damaging stimuli [89]. In addition to myonuclei, single-nucleated populations of cells, of which the best described are satellite cells, also populate skeletal muscle [90]. Satellite cells are muscle precursor cells (i.e., adult stem cells) that are quiescent unless induced to divide by external stressors, such as contraction-induced or injury-induced muscle damage [90]. When induced to divide, satellite cells divide asymmetrically, with one daughter cell incorporating into the damaged muscle fiber and the other daughter cell returning to replenish the satellite cell pool [90]. Skeletal muscle telomere dogma states that when a muscle precursor cell is induced to divide and incorporate into the muscle fiber, the new nuclei will have the shortest telomeres in that fiber owing to the fact that they originate from precursor cells that had divided prior to becoming incorporated [89, 91–93]. With increased regenerative pressures on skeletal muscle, a greater number of nuclei with short telomeres would be present and thus the muscle fiber in total would have shortened telomeres, as is the case for some muscular dystrophies (i.e., Duchenne's muscular dystrophy; [94]).

Muscle contraction, such as heavy-resistance-type exercise, is known to cause injury to skeletal muscle and thus is a stimulus for satellite cell proliferation. Recently, long-duration and high-intensity endurance exercise was shown to cause satellite cell replication in skeletal muscle [95]. Since exercise can result in muscle damage and proliferation of satellite cells, and telomere length shortens with cell division, exercise may cause telomere shortening in skeletal muscle tissue of highly active individuals. This provides support for the inverted U hypothesis for the relationship between exercise and telomere length, with extreme exercise resulting in cellular damage. Using the logic that exercise may be a proliferative stress to skeletal muscle, several groups have investigated the role that physical activity (endurance training and resistance exercise in particular) may play in skeletal muscle telomere biology (Table 1).

Two studies by the same group have investigated the effects of long-term endurance training and found that exercise may cause telomere shortening in skeletal muscle of chronically trained individuals. Though the sample size was small, Collins et al. [76] investigated skeletal muscle telomere length by comparing healthy athletes to chronically overtrained athletes with fatigued-myopathic syndrome (FAMS) who were matched for age and training volume. Despite similar training volumes the symptomatic group had reduced athletic performance, decreased ability to tolerate high-volume training, and excessive muscular fatigue during exercise [76]. All of these symptoms may be indicative that the FAMS athletes had a reduced capacity to repair skeletal muscle damage. The FAMS athletes had shorter telomeres compared to healthy athletes, indicating that the overtrained athletes may have induced greater proliferative stress on satellite cells compared to the healthy athletes. In another study, Rae et al. [73] characterized skeletal muscle telomeres from a large number of healthy endurance trained individuals compared to sedentary individuals. They observed no difference between groups for mean telomere length, but in the endurance-trained group, telomere length was inversely correlated to years and hours of training [73]. This indicates that endurance training could be a replicative stressor to satellite cells in lower limb skeletal muscle or that long-term exercise resulted in other telomere shortening stressors such as excess ROS. One limitation of this study was the quantification of minimal telomere lengths from TRF gels; since the shortest telomeres are likely lost using this method, the authors may have actually underestimated the number of short telomeres in the athletes. A different method that determines the proportion of short telomeres in a sample would provide clarification on the effect of endurance exercise on skeletal muscle telomere length. Importantly, future research should determine how different frequencies, intensities, and durations of exercise result in different proliferative demands on skeletal muscle as indicated by a shift in the proportion of short telomeres.

Since heavy-resistance exercise is a well-known muscle damaging and satellite cell proliferative stimulus, researchers have investigated the effect of chronic resistance training on telomere length in skeletal muscle. Kadi et al. [71] compared long-term competitive weight lifters to healthy age-matched active subjects. There was not an overt difference in telomere length between the resistance-trained and healthy active individuals; however, there was a negative correlation between individual records (i.e., heaviest weight lifted in a particular exercise) and minimal telomere length, indicating that a heavier load was correlated with shorter telomeres in skeletal muscles and was potentially a regenerative stress on the muscle [71]. The authors hypothesized that the shorter telomeres were due to greater satellite cell proliferation resulting from the contraction-induced muscle fiber damage. These data from skeletal muscle are similar to the inverted U phenomena observed in immune cells, in that moderate levels of endurance training or resistance training may maintain or not change telomere length, while extreme exercise levels may result in telomere shortening potentially due to increased cellular proliferation.

Not all data in skeletal muscle indicate that telomere shortening occurs with physical activity. Ponsot et al. [72] investigated telomere length in healthy physically active older men and women and observed that telomere length (both mean and minimum) was similar in active and sedentary individuals, leading to the conclusion that moderate physical activity is not a proliferative stress on skeletal muscle tissue. Two limitations of this study were that the subjects were only in two specific age groups (i.e., young versus old) and not across the age spectrum, and the activity levels were low and not representative of the full activity spectrum. These data indicate that moderate activity levels likely do not cause skeletal muscle damage, do not result in an excess proliferative demand, and do not shorten telomeres. Thus, moderate activity would appear to maintain telomere length with age. These data provide support for the inverted U hypothesis, that if the exercise stimulus is not causing cellular damage, telomere length should be maintained. Building on these data, a recent, small cross-sectional study compared skeletal muscle telomere lengths in young active and sedentary and older active and sedentary individuals. Osthus et al. [70] observed that telomere length in the older active group was longer than that in the older inactive group and that there was a positive correlation between maximal oxygen consumption and telomere length. These data seem to conflict with the current literature in that all other studies have either reported no difference or a small decrease in skeletal muscle telomere length in active individuals. Further, these data would seem to indicate that telomeres were elongated (either by telomerase or another mechanism) in the muscles of active individuals since typically telomere length would be constant with age in skeletal muscle due to its low turnover rate. Thus, these controversial data need to be confirmed in a larger sample size and with a longitudinal study.

In summary, the data for how exercise and physical activity influence telomere biology in human skeletal muscle are mixed and need clarification with longitudinal experiments. The effect of exercise on skeletal muscle telomere length is likely directly linked to the proliferative demand the exercise places upon the skeletal muscle. Thus, the more damaging the exercise stimulus (either endurance- or resistance-type exercise) to the muscle fibers and the greater the proliferative demand upon the muscle stem cells, the faster the rate of skeletal muscle telomere shortening. Furthermore, the longer the exercise duration, both in terms of length of individual exercise bouts and years of training an individual performs, the greater the observed decrease in telomere length. Another potential mechanism to consider is the antioxidant capacity of the skeletal muscle, which could be overwhelmed by contraction-induced reactive oxygen species (ROS) and result in damage to telomere DNA and the stochastic loss of telomere length, as has been shown in other tissues exposed to ROS [96–100]. Furthermore, given the recent findings that exercise may actually elongate telomeres in skeletal muscle from active individuals, mechanisms for how this could be occurring (e.g., telomerase activity) should be explored. The relationship between exercise, physical activity, and skeletal muscle warrants further investigation, especially considering the recent controversial findings that telomere length in skeletal muscle may shorten with age at a similar rate to other proliferative tissues such as immune cells [101].

*Are Telomere Lengths Synchronized across Tissues?* Telomere length in mammalian species is tissue-specific [102, 103]. The standard model in the field is that telomeres in proliferative tissues (e.g., immune cells, intestinal epithelial cells) shorten with age [11], while in low-turnover tissues such as skeletal muscle [89], telomere length is constant over time; thus, telomere length is directly linked to proliferative history of the tissue. This indicates that the rate of telomere shortening is tissue-specific and directly dependent upon proliferative demands and rate of damage accumulation and repair in each tissue. In contrast to this long-held hypothesis, a recent report showed that the rate of telomere shortening in human lymphocytes was similar to the rate of telomere shortening in skeletal muscle and other low turnover tissues [101]. Daniali et al. [101] collected tissues (skin, immune cells, fat, and skeletal muscle) from 87 individuals across the age span 19–77 years and measured telomere length. The major finding of their study was that the rate of age-dependent telomere shortening was similar across the tissues studied despite different replicative dynamics of leukocytes, skeletal muscle, skin, and fat. As opposed to previous studies, Daniali et al. [101] observed skeletal muscle shortening with age, an unexpected finding that they attributed to their large sample size. This indicates that telomere lengths across tissues may be “synchronized” and that the rate of leukocyte telomere length shortening predicts the rate of skeletal muscle telomere shortening with age.

The data from the skeletal muscle telomere and exercise literature seem to directly conflict with Daniali et al.'s [101] data. According to the “synchrony hypothesis,” telomere length in the peripheral blood cells should predict telomere length in other tissues; however, the majority of the literature has not documented an age-related change in skeletal muscle telomere length, while age-related decreases in immune cell telomere length have been widely reported [61, 104, 105]. Moreover, several reports have documented the potential for endurance exercise to significantly shorten telomeres in skeletal muscle while maintaining telomere lengths in other tissues [42, 43, 63, 73, 76]. Reconciliation of whether or not telomere length responds similarly across tissues (as would be expected from the telomere synchrony hypothesis), or if telomere length responds to exercise in a tissue-specific fashion, is an important area of future research.

## 5. Telomerase and Shelterin Response Exercise in Humans

Few reports have described the effects of physical activity, exercise training, or acute exercise on telomerase, shelterin, and other telomere-associated proteins [63, 74]. Of those studies, most have compared immune cells between chronically trained individuals and sedentary individuals. The results of these studies have been mixed, showing either a slight increase or no difference in telomerase activity or expression of shelterin components in the trained groups. There are no studies that we are aware of investigating the effects of a single bout of endurance exercise on telomerase activity in immune cells.

Strenuous exercise is a known proliferative stress for immune cells [106]. Mitogen stimulation of immune cells increases telomerase activity in T cells [107], suggesting that telomerase activity may be increased in long-term exercise-trained individuals in order to maintain telomere length following the repeated proliferative stress of strenuous exercise. Ludlow et al. [43] described a potential gene-environment interaction between physical activity level and a TERT promoter polymorphism that is associated with telomere length and telomerase enzyme activity [108, 109]. Individuals in the highest quartile of physical activity and carrying a specific TERT promoter genotype (rs2735940, C-1327T, TT genotype) were observed to have greater telomerase enzyme activity in PBMCs, thus demonstrating an association between physical activity level, TERT genotype, and telomerase activity [43]. These data were collected from a small cohort, but the association is interesting in that not only did those individuals with the particular genotype exhibit greater telomerase activity, but they also had PBMCs with short telomeres. Moreover, telomerase has been shown to be recruited preferentially to the shortest telomeres [77, 110]. Thus, if telomerase is preferentially recruited to the shortest telomeres in cells of exercise-trained individuals, exercise may prevent the induction of senescence by maintaining the shortest telomeres in these immune cells, thereby slowing an aging phenotype.

In support of this hypothesis, Werner et al. [63] showed that older athletes have immune cell telomerase enzyme activity similar to younger individuals and greater than age-matched sedentary individuals. These data support the hypothesis that telomerase may be part of the adaptive response to exercise training and could be a biomarker of improved physical health [43, 50]. In addition, while Laye et al.'s [74] study did not find a change in telomere length following 7 marathons in 7 days, they did observe increased gene expression of both DNA damage repair proteins and shelterin components in skeletal muscle and immune cells. Taken together these data indicate that exercise training is associated with a telomere-protective phenotype in both leukocytes and skeletal muscle; however, the adaptive mechanisms surrounding telomerase and shelterin may be different between tissues and depend upon the training status and age of the individuals. Future studies will be needed to clarify the exact mechanisms of how exercise results in a telomere-protective environment in specific tissues.

## 6. Mouse Telomere Length Response to Exercise

Since human telomeres are slow to shorten (i.e., over many years), groups have turned to model organisms, such as rodents, to explore the relationship between exercise and telomere dynamics. However, rodents require special considerations when studying telomere biology. In this section, we discuss the positives, negatives, and caveats to using rodents for telomere-related studies and also describe the work that has been completed using mice as model organisms in this area of investigation.

### 6.1. Mouse Telomere Biology Review and Subsequent Caveats to Using Rodents

Though the biology of telomere mechanics is similar between humans and rodents, human telomeres are typically only 7–15 kilobases in length [18], while rodent telomeres are much longer (20 to 50 kilobases and up to 150 kilobases depending on inbreeding status and strain; [111]). Furthermore, humans and other short telomere mammals utilize telomere length as a tumor-suppressive mechanism, while long telomere mammals, such as rodents, do not [102]. Another important difference to consider in rodents is that the majority of somatic tissues are telomerase positive and that the mouse TERT gene is regulated differently than the human TERT gene [112]. Despite these differences, age-related telomere shortening does occur and the shelterin proteins are conserved in rodents, thus making studying the response to environmental stressors in rodents relevant to human biology. To reduce the long telomere bias that rodent studies introduce, rodents with shorter telomeres have been employed, with the most common models being strains with naturally occurring short telomeres (e.g., wild-derived inbred strains such as *mus musculus cataneous*, CAST/Ei) or models where gene disruption of telomerase components, mTERT and mTERC, has been performed [113–115].

Naturally occurring shorter telomere rodents, such as CAST/Ei mice, are typically wild derived, more recently inbred, and have telomere lengths (15–20 kb) much closer to those of humans (7–15 kb) [42, 111, 116]. Several studies have used the CAST/Ei animals in an attempt to more accurately model the response of telomeres and telomere length regulating proteins to environmental stress (e.g., exercise) and oxidative stress [42, 111, 116].

Another way to induce shorter telomere lengths in rodents is by knocking out either the protein catalytic subunit (mTERT) or the RNA component (mTERC) of telomerase. These models are unique in that the first generation of knockout animals retain wild-type telomere lengths while offspring generations three through six display shorter telomere lengths and aging phenotypes compared to age-matched wild-type controls [114]. In addition, the telomerase genetic manipulations have been developed on the CAST/Ei background, potentially providing a model that is even more similar to human telomere biology [115]. Thus, several rodent models are available to study the effects of environmental stressors on telomere length and telomere biology, but special caution must be used when interpreting and extrapolating the results to humans.

### 6.2. Effect of Exercise Training on Telomere Length in Rodent Models

To date only two groups have investigated the effect of long-term exercise training on telomere length in rodent models [41, 42, 63, 117]. Werner et al. [117] investigated the effect of 6-months of exercise training on telomere length in leukocytes and heart muscle of C57BL/6 mice (long telomeres; ~50 kb), as well as in several knockout and transgenic models, in order to delineate a mechanism of telomere protection in response to exercise. In both tissues following either 3 weeks or 6 months of voluntary wheel running activity, no effect on telomere length was observed compared to controls. To test if age-related telomere shortening was occurring in these animals the authors aged sedentary animals for 18 months and were able to detect telomere shortening in both leukocytes and left ventricular heart muscle. Thus, these first important analyses highlight the need for longer-duration-studies to delineate the effect of exercise on telomere length in rodents.

Ludlow et al. [42] performed a similar study but in the CAST/Ei mouse strain. Three groups of animals were investigated: 8-week-old sedentary, one-year-old sedentary, and one-year-old animals that had access to a voluntary running wheel for 44 weeks. Telomere length was assessed in liver, heart, and skeletal muscle tissues. Significant age-related telomere shortening in the heart and liver of the one-year old animals was attenuated by voluntary wheel running. In skeletal muscle, significant telomere shortening was observed in the chronic exercise group compared to both the sedentary young animals and the one-year-old animals. These data indicate that exercise not only affects telomere biology in leukocytes but also in cardiac muscle, skeletal muscle and liver, albeit with tissue-specific effects. These tissue-specific responses are likely related to differences in the proliferative demands placed on the individual tissues by the exercise stimulus, as well as differences in antioxidant capacity between the tissues. Future research is needed to determine if proliferation and oxidative stress are responsible for these tissue-specific responses.

### 6.3. Effect of Exercise on Telomere Length-Maintaining Proteins in Rodent Models

Several groups have investigated how exercise may alter the expression of telomere-related proteins in rodent tissue. Werner et al. [63, 117] performed a thorough series of experiments to elucidate how exercise produced adaptations in cells that made them more resistant to environmental stressors, specifically investigating the role of telomeres and telomere-related proteins in left ventricular, aortic, vascular, and immune tissues. Investigating short-term training effects (21 days of voluntary wheel running) in C57BL/6 mice, they observed that TERT protein and TRF2, Ku70, and Ku80 mRNA expression levels were increased compared to sedentary controls. These data indicate that short-term exercise training in rodents is associated with increases in both telomere length and senescence protective expression profiles. To determine if the effects of exercise for 21 days were dependent upon TERT protein, TERT knockout animals were given access to a running wheel for three weeks. It was observed that the effects of exercise on TRF2, p16, Chk2, and p53 were not present in the exercised knockout animals compared to wild-type exercised animals [63, 117]. These data provide evidence that TERT protein may be needed for the beneficial adaptation of exercise on telomere-related proteins, indicating that in rodent tissues TERT may have extratelomeric functions, such as acting as a transcription factor or part of a chromatin remodeling complex [118]. These data provide substantial evidence that exercise and physical activity can result in a cellular environment that is protective against shortened telomeres and subsequently protective against aging phenotypes in heart, vascular, and immune cells.

In a different strain of mice (CAST/Ei), Ludlow et al. [42] observed tissue-specific responses of shelterin and telomerase to 44 weeks of voluntary wheel running. In skeletal muscle, exercise resulted in shortened telomeres, but increased telomerase enzyme activity. In addition, there was an age-associated increase in skeletal muscle TRF1 protein levels that was attenuated by exercise. In cardiac muscle, exercise attenuated the age-related reduction in shelterin gene expression, while in liver tissue no significant changes were observed. These data indicate that while exercise is beneficial to all three tissues, the adaptive response of telomere length regulating proteins is tissue-specific.

Overall these data indicate that telomere-binding proteins and telomerase adapt to exercise training in a tissue-specific fashion. Some tissues upregulate telomerase enzyme activity, while other tissues seem to alter the expression of shelterin components and DNA damage response and repair genes. These data indicate that exercise likely results in tissue-specific adaptation of the telomere maintenance pathways; however, regardless of tissue-specific effects, it is important to note that exercise results in a phenotype that is teloprotective in most tissues studied to date.

## 7. Signaling Mechanisms Associated with the Adaptive Response of Telomere Length-Regulating Proteins

Elucidating the mechanisms of how telomere length is preserved or lost following exercise is important for understanding how telomeres respond to physiological stressors. To date most mechanistic studies on exercise have focused on the stress response and growth/cellular proliferation pathways. Only three studies have investigated exercise-specific signaling mechanisms associated with altered telomere biology, all of which have been performed in rodent cells and tissues [41, 63, 117]. TERT, IGF-1, eNOS, and AKT were identified as being important in signal transduction of the exercise-induced telomere protective phenotype [63, 117]. In addition, p38MAPK was shown to regulate the gene expression of *Trf1* following acute exercise in rodent skeletal muscle [41]. These data indicate that more work is needed to fully elucidate the signaling mechanisms of exercise-induced telomere protection and point to the stress response and growth/cellular proliferation pathways as high-priority candidates for future studies.

## 8. Future Directions

Exploration of the effect that physiological stressors such as exercise and exercise training have on the structure-function relationship of the genome is fertile ground. Researchers should consider the following five directions of importance for future research: (1) determining whether or not telomere length shortens at equal rates across somatic tissues in response to exercise training; (2) if an inverted U relationship exists between physical activity and telomere length; (3) the tissue-specific functional consequences of short telomeres in trained versus untrained individuals; (4) the role of oxidative stress and inflammation during and following exercise and the effects on telomere biology; and (5) the specific pathways (e.g., stress response, growth, and proliferation) that cause the adaptation and response of telomerase and shelterin to exercise and how these adaptations result in altered telomere length.

## 9. Conclusions

Numerous studies have implicated a telomere-protective phenotype induced by moderate levels of physical activity, indicating an important cellular adaptation that may slow the onset of symptoms or prevent certain age-related diseases. In contrast, several lines of evidence in both immune cells and skeletal muscle indicate that telomeres may actually shorten in response to long-term high-intensity endurance training. As such, the tissue-specific response of telomeres should be investigated, with specific consideration given to the proliferative demands placed on the tissue by the exercise stimulus and the antioxidant capacity of the individual tissues. Understanding how telomeres adapt on a tissue-specific basis and if immune cells are predictive of the adaptive response of other tissues is a necessary next step in this field. Additionally, determination of the type, time, intensity, and frequency of exercise that results in an excess proliferative demand on immune and skeletal muscle tissues and results in loss of telomere length is important. A multidisciplinary approach must be taken to tackle these important questions and to further solidify telomere length as a useful biomarker in monitoring the long-term effects of environmental and physiological stressors, such as exercise training.

## Figures and Tables

**Table 1a:** (a)

Positive association of physical activity with telomere length				
Author	Study design	Subjects (*N*)	Tissue	Telomere length method
Cherkas et al. [61]	Cross-sectional	Twin cohort (2401)	Leukocytes-PBMCs	T/S qPCR
Werner et al. [63]	Cross-sectional	Young sedentary (26), young athletes (25), older sedentary (26), and older athletes (25)	Leukocytes	QFISH and T/S qPCR
Mirabello et al. [60]	Cross-sectional	Prostate cancer cases (612) versus age-matched controls (1049)	Leukocytes	T/S qPCR
Simpson et al. [59]	Longitudinal	Endurance trained men (9)	Sorted populations of PBMCs	T/S qPCR
Puterman et al. [45]	Cross-sectional	Postmenopausal women (63)	Leukocytes	T/S qPCR
LaRocca et al. [62]	Cross-sectional	Young sedentary (15), young athletes (10), older sedentary (15), and older athletes (17)	Leukocytes	Southern blot TRF
Krauss et al. [64]	Cross-sectional	Heart and Soul population (944)	Leukocytes	T/S qPCR
Kim et al. [58]	Cross-sectional	Postmenopausal women (44)	Leukocytes-PBMCs	T/S qPCR
Du et al. [57]	Cross-sectional	Nurse's health study (7,813)	Leukocytes	T/S qPCR
Osthus et al. [70]	Cross-sectional	Young sedentary (5), young athletes (5), older sedentary (5), and older athletes (5)	Skeletal muscle	T/S qPCR

Ref: reference. T/S qPCR: the ratio of telomere PCR value to single-copy gene value derived from quantitative PCR. TRF: terminal restriction fragment analysis. QFISH: quantitative fluorescence in situ hybridization with a telomere probe. *N*: number of subjects.

**Table 1b:** (b)

No association of physical activity with telomere length				
Author	Study design	Subjects (*N*)	Tissue	Telomere length method
Woo et al. [69]	Cross-sectional	65 years or older Chinese men and women (4000)	Leukocytes	T/S qPCR
Ornish et al. [50]	Longitudinal	Prostate cancer patients (30)	Leukocytes	T/S qPCR
Song et al. [68]	Cross-sectional	Diverse population (103)	Leukocytes	T/S qPCR
Mason et al. [67]	Randomized trial	Postmenopausal women (439)	Leukocytes	T/S qPCR
Mathur et al. [66]	Cross-sectional	Marathon athletes (17) versus matched individuals (15)	Lymphocytes and granulocytes	T/S qPCR
Kadi et al. [71]	Cross-sectional	Resistance trained strength athletes (7) versus active individuals (7)	Skeletal muscle	Southern blot TRF
Ponsot et al. [72]	Cross-sectional	Diverse population (42)	Skeletal muscle	Southern blot TRF
Rae et al. [73]	Cross-sectional	Healthy endurance runners (18) versus sedentary age-matched (19)	Skeletal muscle	Southern blot TRF
Laye et al. [74]	Longitudinal	Marathon athletes (8)	Skeletal muscle and leukocytes	T/S qPCR

Ref: reference. “Diverse population” refers to a sample with a broad age range with both males and females of multiple races. T/S qPCR: the ratio of telomere PCR value to single-copy gene value derived from quantitative PCR. TRF: terminal restriction fragment analysis. *N*: number of subjects.

**Table 1c:** (c)

Inverted U relationship of physical activity with telomere length				
Author	Study design	Subjects (*N*)	Tissue	Telomere length method
Ludlow et. al. [43]	Cross-sectional	Population, 50–70-year-olds (69)	Leukocytes-PBMCs	T/S qPCR
Savela et al. [75]	Cross-sectional	Men (782)	Leukocytes	Southern blot TRF
Collins et al. [76]	Case-control	FAMS athletes (13) versus healthy endurance athletes (13)	Skeletal muscle	Southern blot TRF

Ref: reference. “Diverse population” refers to a sample with a broad age range with both males and females of multiple races. T/S qPCR: the ratio of telomere PCR value to single-copy gene value derived from quantitative PCR. TRF: terminal restriction fragment analysis. *N*: number of subjects. FAMS: fatigued athlete myopathic syndrome.
